# Altered proteome of high-density lipoproteins from paediatric acute lymphoblastic leukemia survivors

**DOI:** 10.1038/s41598-019-40906-x

**Published:** 2019-03-12

**Authors:** Maryse Fournier, Eric Bonneil, Carole Garofalo, Guy Grimard, Caroline Laverdière, Maja Krajinovic, Simon Drouin, Daniel Sinnett, Valérie Marcil, Emile Levy

**Affiliations:** 1 Research Centre, Sainte-Justine University Hospital Health Center, Université de Montréal, Montreal, H3T 1C5, Quebec, Canada; 2 Department of Nutrition, Université de Montréal, Montreal, H3T 1C5 Quebec, Canada; 3 Institute of Research in Immunology and Cancer, Université de Montréal, QC H3C 3J7 Montréal, Canada; 4 Department of Pediatrics, Université de Montréal, Montreal, H3T 1C5 Quebec, Canada

## Abstract

Acute lymphoblastic leukemia (ALL) is the most frequent malignancy in children. With the use of more modern, efficient treatments, 5-year survival has reached more than 90% in this population. However, this achievement comes with many secondary and long-term effects since more than 65% of the survivors experience at least one severe complication, including the metabolic syndrome and cardiovascular diseases. The main objective of the present work was to characterize the composition of HDL particles isolated from pediatric ALL survivors. HDLs from 8 metabolically healthy ALL survivors, 8 metabolically unhealthy ALL survivors and 8 age- and gender-matched controls were analyzed. The HDL fraction from the survivors contained less cholesterol than the controls. In addition, proteomic analyses revealed an enrichment of pro-thrombotic (e.g., fibrinogen) and pro-inflammatory (e.g., amyloid A) proteins in the HDLs deriving from metabolically unhealthy survivors. These results indicate an alteration in the composition of lipid and protein content of HDL from childhood ALL survivors with metabolic disorders. Although more work is needed to validate the functionality of these HDLs, the data seem relevant for survivor health given the detection of potential biomarkers related to HDL metabolism and functionality in cancer.

## Introduction

Childhood cancers are rare, representing only 0.5–1% of all the cancers diagnosed in Canada. Despite the remarkable overall survival rate (83%, 5-years post-diagnosis), cancer remains the first cause of death from illness in the pediatric population^1^. Leukemias are the most common pediatric malignancy (32%) with acute lymphoblastic leukemias (ALL) accounting for 80% of all leukemia cases^1^. The 5-year survival rate of ALL has risen from 0% in the 1950’s^2^ to the astounding level of 91%^1^. This was made possible with the use of intense regimens of effective but extremely toxic therapies. It is a rising concern to administer such dangerous treatments in a crucial developmental stage, which may affect the health of the young survivors many years later. Indeed, more than 65% of the childhood cancer survivors have at least one long-term complication, which are severe and often life-threatening^3–6^. One of these complications regularly found in cancer survivors is the metabolic syndrome, e.g. a cluster of obesity, insulin resistance, hypertension, and dyslipidemia. Its prevalence may vary from 6–55% in survivors, depending on many factors such as the age of onset, the type of cancer, the kind of treatments, the duration of follow-up and others^7,8^. In fact, hematologic cancer survivors are twice more likely to develop the metabolic syndrome than their siblings or the general population^9,10^.

HDL particles are widely known in view of their anti-atherosclerotic properties. These features are not only due to their ability to promote reverse cholesterol transport^11^, but are likely the result of many functions, which help maintain metabolic health because of their antioxidant^12,13^, anti-apoptotic, anti-inflammatory^14–16^, anti-hypertensive^17,18^ and anti-thrombotic^19^ capacities. These numerous functions are made possible by the large variety of protein and lipid moieties within the HDL fraction. Since proteomics allow high-throughput analyses for the detection, identification and functional investigation of proteome, it seems reasonable and appropriate to use this large-scale technique to scrutinize the variations of the multitude of proteins composing the HDL^20^. Not only did proteomics generally identify ~100 individual proteins as part of the HDL^21^, they also revealed dysfunctional HDLs in many pathologies, including kidney disease^22,23^, type 1 diabetes^1,24^, type 2 diabetes^12^, cardiovascular diseases^17,25–27^, lupus erythematous^28^ and rheumatoid arthritis^9^. When investigated, these dysfunctions where often linked to a change in the HDL’s proteome such as a shift towards more inflammatory proteins^15,25,26^ and post-translational modifications of the proteins^1^. Intriguingly, very limited information is available on the composition and functionality of HDL in ALL survivors even though the latter develop metabolic syndrome. Our objective was to describe the global HDL composition and further investigate the changes in the proteome of HDLs isolated from childhood ALL survivors.

## Results

### Study participants

The present study includes a total of 24 subjects (50% males): 8 healthy subjects (Controls), 8 metabolically healthy ALL survivors (Healthy), and 8 metabolically unhealthy ALL survivors (Unhealthy). The participants were matched for age and gender. The anthropometrics data are presented in Table 1, the metabolic characterization is presented in Table 2, while the lipid profiles of the participants are presented in Table 3. The analyses on the therapies administered to the patients concern 14 participants due to missing values in 2 cases.

**Table 1:** Anthropometric characteristics and treatment details of the participants.

	Controls	ALL Survivors		
		All	Healthy	Unhealthy
**N** (male)	8 (4)	16 (8)	8 (4)	8 (4)
**Age** (years)	24.2 ± 2.0	24.3 ± 1.1	23.3 ± 1.6	25.4 ± 1.6
**BMI** (kg/m^2^)	23.5 ± 0.8	25.3 ± 1.6	22.3 ± 1.0	28.2 ± 2.7
**Waist circumference (cm)**				
Men	87.0 ± 2.8	86.4 ± 4.9	79.1 ± 7.4	93.8 ± 4.6
Women	80.6 ± 2.7	88.1 ± 7.7	78.6 ± 2.9	97.5 ± 14.5
All	83.8 ± 2.2	87.2 ± 4.4	78.8 ± 3.7	95.6 ± 7.1^#^
**Age at diagnosis** (years)	NA	8.5 ± 1.3	10.9 ± 1.8	6.2 ± 1.7^#^
**Event-free remission** (years)	NA	13.6 ± 1.6	10.2 ± 1.1	17.1 ± 2.6
**Treatments**				
Chemotherapy only	NA	**2**	**2**	**0**
Without cardioprotection	NA	1	1	0
With cardioprotection	NA	1	1	0
Radiotherapy only	NA	0	0	0
Chemotherapy + radiotherapy		**12**	**6**	**6**
Without cardioprotection		5	1	4
With cardioprotection		7	5	2
**Cranial radiotherapy dose (Gy)**				
0	NA	2	2	0
12	NA	6	4	2
18		6	2	4
**Protocol administered**				
DFCI 1987-01	NA	1	0	1
DFCI 1991-01	NA	2	0	2
DFCI 1995-01	NA	3	2	1
DFCI 2000-01	NA	5	4	1
DFCI 2005-01	NA	3	2	1
**Risk group**				
Standard risk	NA	12	7	5
High risk	NA	4	1	3
**Corcorticosteroids** (mg/m^2^)	NA	11921	13573	10269
**Active smoking**	0	3	1	2

Data on anthropometrics were collected on ALL survivors (n = 16) and age- and gender-matched controls (n = 8). All survivors were stratified in 2 groups according to their metabolic status as described in Methods (n = 8/group).

NA, non-applicable.

Results are presented as Mean ± SEM. BMI: body mass index, DFCII: Dana-Farber Cancer Institute, Gy: Gray; ^#^p < 0.05 vs Healthy.

**Table 2:** Metabolic characterization of the study participants.

	Controls	ALL Survivors		
		All	Healthy	Unhealthy
**BP (mmHg)**				
Systolic	—	124 ± 4	117 ± 4	130 ± 7
Diastolic	—	72 ± 3	66 ± 3	77 ± 5
**FBG** (mmol/L)	5.1 ± 0.1	4.9 ± 0.1	4.8 ± 0.1	4.9 ± 0.2
**OGTT (mmol/L)**				
30 min	—	8.5 ± 0.4	8.1 ± 0.5	8.9 ± 0.6
60 min	—	7.8 ± 0.6	6.5 ± 0.5	9.2 ± 0.9^#^
120 min	—	5.9 ± 0.4	5.4 ± 0.3	6.4 ± 0.8
**MDA** (pmol/ml)	298 ± 49	685 ± 95^*^	692 ± 172	678 ± 96
**CRP** (mg/L)	—	1.8 ± 0.5	1.1 ± 0.5	2.5 ± 0.7^#^
**Number of MetS criteria**				
0	8	8	8	0
1	0	1	0	1
2	0	6	0	6
3	0	0	0	0
4	0	1	0	1

Data on metabolic status were collected on ALL survivors (n = 16) and age- and gender-matched controls (n = 8). ALL survivors were stratified in 2 groups according to their metabolic status as described in Methods (n = 8/group). Mets was defined per the NCEP ATP III (SOURCE), with the following cut-offs: (i) waist circumference >88 cm for women and >102 cm for men, (ii) HDL-C <1.29 mmol/L for women and <1.03 mmol/L for men, (iii) systolic blood pressure >130 or diastolic >85 mmHg, (iv) fasting blood glucose >6.1 mmol/L. Results are presented as Mean ± SEM. BP: blood pressure, CRP: C-reactive protein, FBG: fasting blood glucose, MDA: malondialdehyde, MetS: metabolic syndrome, OGTT: oral glucose tolerance test; *p < 0.05 vs Controls, ^#^p < 0.05 vs Healthy.

**Table 3:** Lipid profile and HDL composition of the study participants.

	Controls	ALL Survivors		
		All	Healthy	Unhealthy
**TG** (mmol/L)	0.90 ± 0.08	1.30 ± 0.36	0.79 ± 0.10	1.82 ± 0.69
**TC** (mmol/L)	4.24 ± 0.15	4.46 ± 0.21	4.10 ± 0.22	4.82 ± 0.32
**LDL-C** (mmol/L)	2.27 ± 0.12	2.59 ± 0.16	2.22 ± 0.15	3.00 ± 0.20**^##^
**HDL-C (mmol/L)**				
Men	1.41 ± 0.11	1.11 ± 0.09	1.29 ± 0.12	0.93 ± 0.03*
Women	1.71 ± 0.08	1.42 ± 0.14	1.76 ± 0.11	1.07 ± 0.05**^##^
All	1.56 ± 0.09	1.26 ± 0.09	1.53 ± 0.12	1.00 ± 0.04**^###^
**Non-HDL-C** (mmol/L)	2.68 ± 0.15	3.20 ± 0.24	2.58 ± 0.14	3.82 ± 0.33*^##^
**Ratio TC/HDL-C**	2.8 ± 0.2	3.8 ± 0.3	2.8 ± 0.2	4.9 ± 0.4***^###^
**% HDL**				
Triglycerides	1.95 ± 0.29	2.55 ± 0.89	1.33 ± 0.23	3.76 ± 1.72
Free Cholesterol	3.93 ± 0.21	3.33 ± 0.12*	3.62 ± 0.09	3.04 ± 0.17***^##^
Esterified Cholesterol	19.66 ± 1.12	17.51 ± 0.56	18.74 ± 0.64	16.28 ± 0.70**^#^
Phospholipids	29.83 ± 1.28	29.00 ± 0.96	29.12 ± 1.20	28.88 ± 1.59
Proteins	44.63 ± 1.55	47.62 ± 1.21	47.20 ± 1.40	48.05 ± 2.06
Weight ratio	0.28 ± 0.02	0.25 ± 0.01*	0.25 ± 0.01	0.25 ± 0.02

Lipid profile and HDL were obtained from ALL survivors (n = 16) and age- and gender-matched controls (n = 8). ALL survivors were stratified in 2 groups according to their metabolic status as described in Methods (n = 8/group). Results are presented as Mean ± SEM. HDL-C: high-density lipoprotein cholesterol; LDL-C: low-density lipoprotein cholesterol; TC: total cholesterol; TG: triglycerides; *p < 0.05, **p < 0.01, ***P < 0.001 vs Controls; ^#^p < 0.05, ^##^p < 0.01, ^###^p < 0.001 vs Healthy.

Although BMI was similar among the groups, the Unhealthy survivors displayed a significantly higher waist circumference than the Healthy survivors (Table 1). The Survivors did not differ in terms of age at diagnosis and time of event-free remission (Table 1). Most of the Survivors (85,7%) received at least 12 Gy of radiotherapy. All the Unhealthy, but only 75% of the Healthy, were subjected to cranial radiotherapy. The Healthy were also better protected against cardiotoxicity: 75% of them received Dexrazoxane (which is a cardioprotective agent) versus 33% of the Unhealthy. Finally, at least one Unhealthy was treated under a version of every protocol (1987-01, 1991-01, 1995-01, 2000-01, 2005-01), while the Healthy received only the 3 latest versions. The proportion of patients at standard and high risk of relapse and diagnosis, and cumulative doses of glucocorticoids were similar amongst groups. Three Survivors (18,8%) reported to be active smokers, and the proportion was not statistically different between groups (Table 1).

The Survivors did not statistically differ as to their blood pressure (Table 2). For glycaemia, the 3 groups have similar fasting glucose concentrations, but the levels of the Unhealthy raised significantly more at 60 min following sugar intake. Importantly, the Survivors displayed higher levels of plasmatic MDA than the Controls (Table 2), and the Unhealthy disclosed a higher CRP level than the Healthy. Per our classification, all the Healthy and Controls displayed 0 component of the metabolic syndrome, whereas all the Unhealthy showed dyslipidemia (low HDL-C), and about 85% had at least 2 components (Table 2).

All the Unhealthy presented with dyslipidemia (Table 3), some of them combining two abnormal lipid components such as low HDL-C (<1.03 for men; <1.30 mmol/L for women) with high TG (>1.7 mmol/L), or high LDL-C (>3.4 mmol/L). Compared to the Healthy and Control groups, the Unhealthy had higher levels of LDL-C and non-HDL-C, a higher ratio of TC/HDL-C, as well as lower levels of HDL-C. TC and TG levels were similar among the groups.

### HDL composition

The composition of the HDL, expressed as the percent of total components, is presented in Table 3. The HDLs from the Survivors showed a significantly lower content of FC compared to those of Controls, with the Unhealthy being even more depleted than the Healthy. EC was also diminished in the Unhealthy. On the other hand, the content in TG, PL, and proteins was similar in the 3 groups. The calculated weight ratio, which gives an indication of the size of the HDL, was higher in the Controls than in the Survivors, thereby indicating a slightly larger size for control HDLs.

### HDL proteome analysis by LC-MS/MS

Although 204 proteins were identified by mass spectrometry (data not shown), only the proteins with at least 2 peptides found in a single sample measurement are presented in Supplementary Table S1. Interestingly, only 31 proteins were commonly identified in all the 24 samples. Inversely, 26 proteins were single-hits, and 46 (34%) were only present in 3 samples or less.

### Functions of the proteins

**Figure 1:**
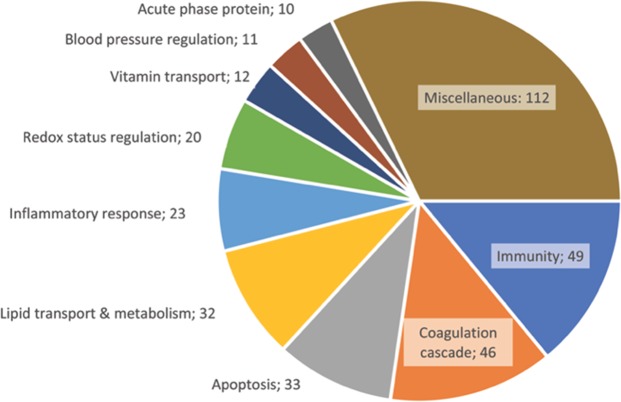
Roles of the different proteins found in the HDL. The HDL function enabled by the detected proteins were defined from the Gene Ontology terms from the UniProt Knowledgebase (n = 10 categories, n = 134 proteins). All the roles that applied for a single protein were considered. Results define the role and the number of proteins involved in this function.

We classified the identified proteins according to their known HDL function, when the information was available. Figure 1 presents a compilation of the different roles of the proteins detected in our study, as sorted out by the Gene Ontology terms from the UniProt KnowledgeBase. We regrouped the terms in categories relating to lipid transport and metabolism, redox status regulation, coagulation cascade, immunity, apoptosis regulation, inflammatory response regulation, vitamin transport, blood pressure regulation and the acute phase proteins. Since some proteins are involved in additional biological pathways, we consequently created the category miscellaneous. Because we considered all the functions for each individual protein, the total exceeds n = 134 proteins. Indeed, 112 proteins have at least one action of little interest, with 30 of these proteins having no interest for us at all. Two apolipoproteins (Apo A-I and Apo E), were the most polyvalent, each fulfilling 9 roles (all except acute phase protein). Apo J and Apo A-II fulfilled 7 roles, while Apo D, angiotensinogen and thrombospondin-1 were players in 6 categories. However, most of the proteins (57,5%) could be classified only in 1–2 categories. Surprisingly, the category lipid transport and metabolism came in the fifth place (23.9%), behind miscellaneous (83,5%), immunity (36.6%), coagulation cascade (34.3%), and apoptosis (24.6%). Of the 31 proteins found in every single sample, the distribution was different: miscellaneous still led with 64.5% of the proteins, but lipid transport and metabolism came in second with 54.8%, followed by immunity, inflammatory response, and coagulation cascade at 45,3%, 32,3%, and 32,3%, respectively.

### Proteins present only in the ALL Survivors

**Figure 2:**
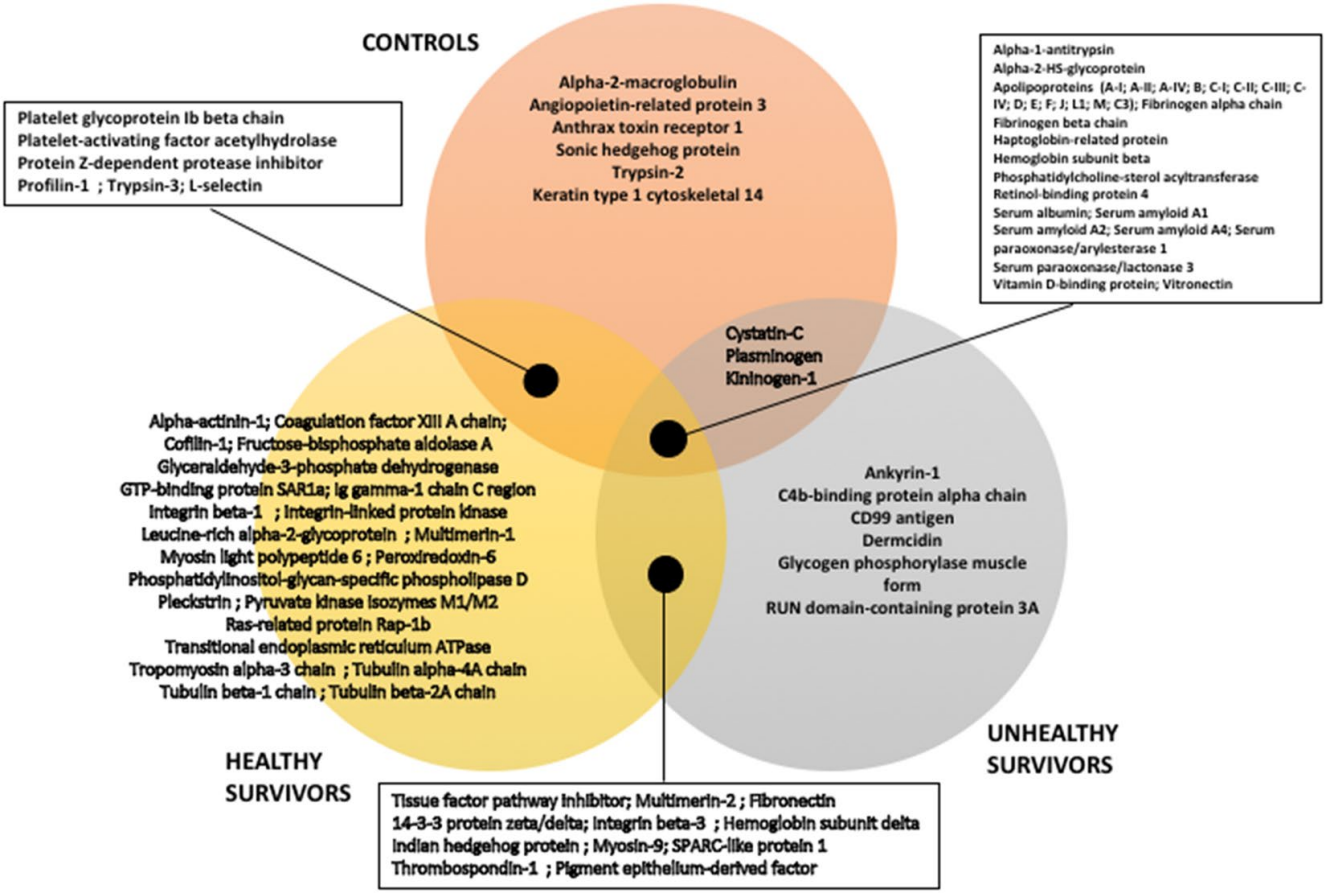
Venn diagram representing the proteins present in the 3 groups.

The proteins present in each group of subjects are illustrated in the Venn diagram, which was constructed to clarify the overlap among protein clusters (Fig. 2). Thirty-nine proteins were found in at least one survivor but were totally absent of the Control group. While 15 of these proteins do not have any known function that relates to the HDL, 7 proteins could be beneficial to the HDL function (since they promote an anti-atherosclerotic action), 15 are detrimental (as they enhance the risks for cardiovascular diseases), and 2 proteins might be both beneficial and detrimental. The beneficial proteins are mostly anti-apoptotic or pro-immunity, with the pigment epithelium-derived factor also being anti-inflammatory, peroxiredoxin-6 being antioxidant and promoting lipid catabolism, and tissue factor pathway inhibitor being anti-thrombotic. 14-3-3 protein zeta/delta and integrin-linked protein kinase are both pro-thrombotic and anti-apoptotic. Finally, 13 proteins promote thrombosis, 4 are pro-apoptotic, and 2 interfere with the transport and metabolism of lipids. The worst proteins are integrin beta-3 (pro-thrombotic, negatively regulating lipid transport and reducing the biosynthesis of LDL-receptor), thrombospondin-1 (pro-apoptotic, pro-inflammatory, pro-oxidant, anti-immunity and pro-thrombotic), and phosphatidylinositol-glycan-specific phospholipase D (pro-apoptotic, negatively regulating TG catabolism and promoting HDL particle clearance). Apparently, the anti-thrombotic capacity of the HDL would be compromised in ALL survivors.

### Proteins missing in all the Survivors samples

On the other hand, only 6 proteins were exclusive to the Control group: alpha-2-macroglobulin, angiopoietin-related protein 3, anthrax toxin receptor 1, sonic hedgehog protein, trypsin-2, and keratin type 1 cytoskeletal 14 (Fig. 2). These proteins have no impact on the regulation of the redox status/blood pressure/inflammation, nor the acute phase response or vitamin transport. Angiopoietin-related protein 3 is considered beneficial as it promotes lipid metabolism and raises HDL-C levels. Sonic hedgehog protein is ambivalent, being anti-apoptotic while negatively regulating the cholesterol efflux. Alpha-2-macroglobulin is detrimental as it is anti-immunity and pro-thrombotic. The last 3 proteins have no known link with the HDL function.

### Proteins absent in the HDLs from Unhealthy survivors

We found 6 proteins present in the HDLs of both the Controls and Healthy, but absent in Unhealthy samples: L-selectin, platelet glycoprotein Ib beta chain, trypsin-3, platelet-activating factor acetylhydrolase, protein Z-dependent protease inhibitor and profilin-1 (Fig. 2). The beneficial proteins L-selectin and protein Z-dependent protease inhibitor promote immunity and have an anti-thrombotic activity, respectively. Platelet-activating factor acetylhydrolase is mostly beneficial (being anti-thrombotic and promoting lipid metabolism) even if it can promote the oxidation of lipoproteins. Platelet glycoprotein Ib beta chain is pro-thrombotic, while trypsin-3 and profilin-1 have no effect on the HDL’s functions of interest.

### Proteins present only in the Unhealthy samples

Finally, the Unhealthy samples displayed 6 proteins that were not found in the participants with 0 metabolic disorder: Ankyrin-1, C4b-binding protein alpha chain, CD99 antigen, dermcidin, glycogen phosphorylase muscle form, and RUN domain-containing protein 3A (Fig. 2). The only 2 proteins with known functions related to the HDL are C4b-binding protein alpha chain and dermcidin, even if dermcidin is mostly contained in the sweat. C4b-binding protein is a potent inhibitor of the complement activation, thus limiting the action of the immune system.

### Protein quantification

**Figure 3:**
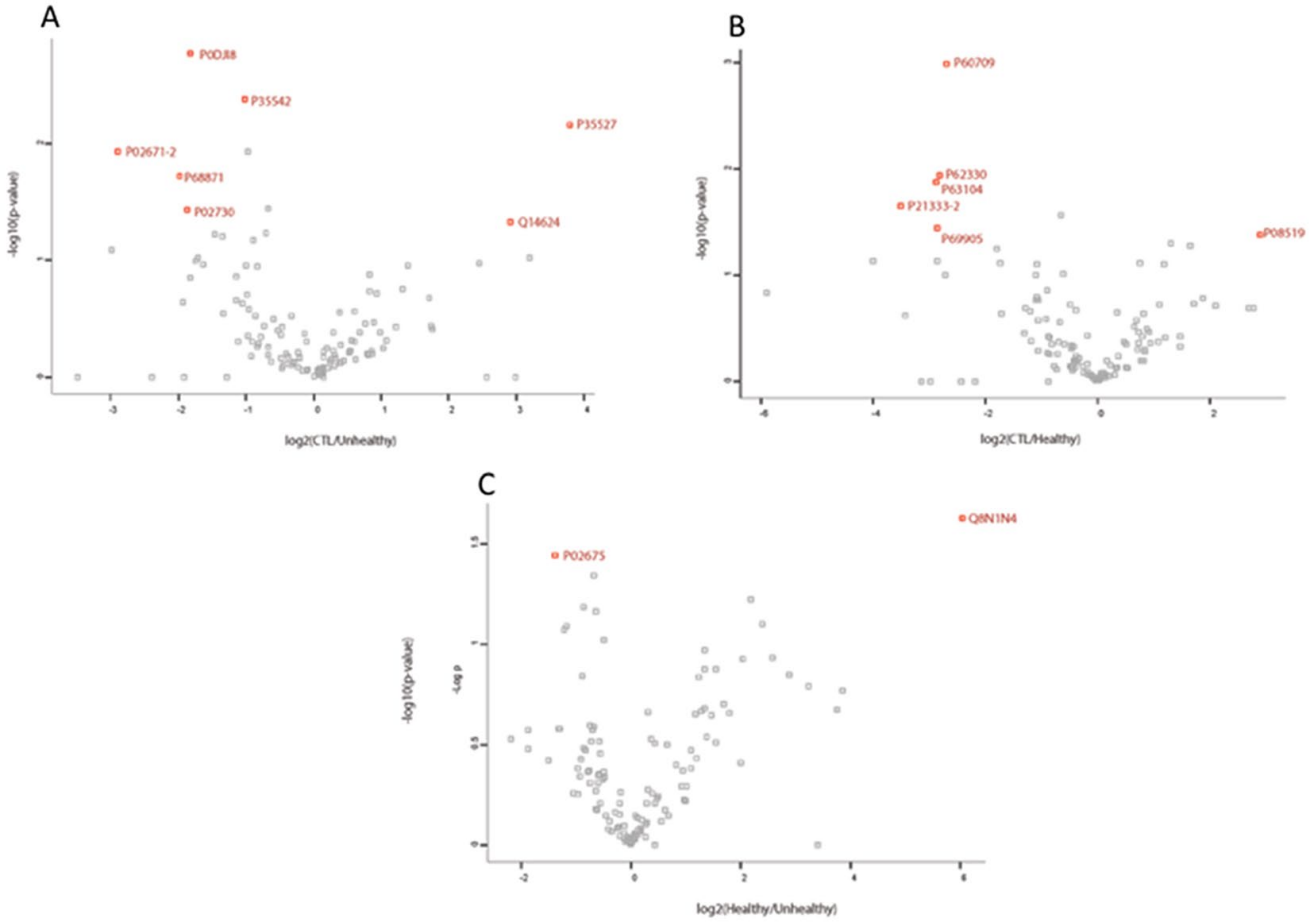
Volcano plot representations of the proteins statistically differently expressed between 2 groups. **(A)** Controls vs Healthy, **(B)** Controls vs Unhealthy, **(C)** Healthy vs Unhealthy. X-axis represents the variation in expression [log_2_(fold change)], y-axis represents the statistical significance [−log(p-value)]. Proteins with coordinates (x, y) where x is <−1 or >1 and y is >1,3 are statistically reproducibly halved or doubled in a group of samples.

To gain insight into the differential HDL proteins detected in the 3 groups, we quantified peptide abundances by measuring precursor peak areas, which is a label-free technique. It is established that the area under the curve of a peptide correlates quite well with the quantity of the protein in the sample^20^. In Fig. 3, the results are presented in the form of Volcano plots, which indicate both the magnitude of the change (log_2_ fold change), x-axis) and the statistical significance (−log10 (p-value), y-axis) defined as the reproducibility of the observation. We considered that a modification in the expression of a protein would have a potential clinical significance provided it was at least halved or doubled compared to the controls, which corresponds to the values inferior to «−1» or superior to «1» on the x-axis. On the y axis, a value > 1, 3 equals a p-value < 0,05. We identified the proteins that corresponded to these two criteria in the different analyses.

When we included the two genders in the analysis, the Unhealthy survivors have more fibrinogen alpha chain, hemoglobin beta chain, SAA1, SAA4 and band 3 anion transport protein on their HDLs than their matched Controls (Fig. 3A). On the other hand, the Controls display more keratin (K1C9) and inter-alpha-trypsin inhibitor 4 than the Unhealthy (Fig. 3B). The Healthy survivors have more actin (cytoplasmic 1), ADP-ribosylation factor 6, 14-3-3 protein zeta/delta, filamin-A and hemoglobin alpha chain than their Controls, while the Controls have surprisingly more Apo (a) on their HDLs. Finally, the Healthy survivors present less fibrinogen beta chain and more keratin (type 2 cytoskeletal 78) than the Unhealthy survivors (Fig. 3C).

When we refined the analysis to focus only on the samples from female subjects, it appears that the Unhealthy survivors are depleted in alpha-1-antitrypsin and enriched in SAA1 and SAA4 versus the Controls (Supplementary Fig. S3A), while there is no difference between the Controls and the Healthy survivors (Supplementary Fig. S3B). Similarly, the male Unhealthy survivors are depleted in indian hedgehog protein and enriched in fibrinogen alpha chain and beta-2-microglobulin versus the Controls (Supplementary Fig. S4A). These Controls have less hemoglobin alpha and beta, as well as less Apo C-I than the Healthy survivors (Supplementary Fig. S4B).

### Proteins with a differential expression among the groups

Besides displaying 13 pro-thrombotic proteins that are totally absent from HDLs of Controls, the HDLs of Unhealthy survivors also overexpressed 4 pro-thrombotic proteins, as well as 2 pro-inflammatory proteins (Fig. 3). When we refined the analyses by gender, we observed a similar tendency: the HDLs of female Unhealthy survivors were enriched in the pro-inflammatory proteins SAA1 and SAA4 versus the Controls. Similarly, the HDLs of male Unhealthy survivors were enriched in fibrinogen alpha chain (pro-thrombotic, pro-hypertensive and anti-apoptotic) versus Controls. These controls have less hemoglobin (alpha and beta, pro-apoptotic but antioxidant) and Apo C-I (anti-lipid metabolism) than the Healthy survivors.

### Biochemical and functional validation of HDL-associated proteins

**Figure 4:**
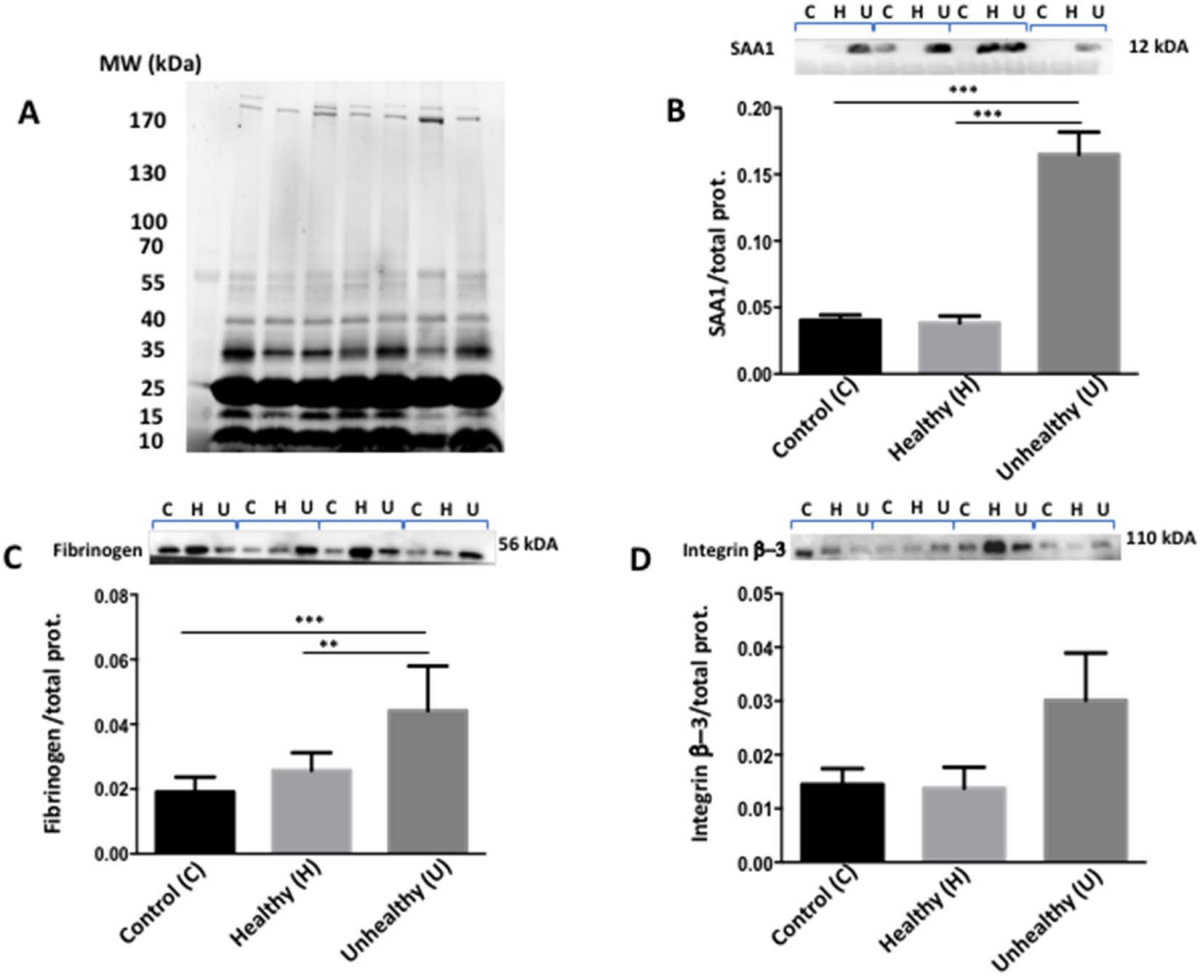
Expression of specific HDL-associated proteins. Proteins from isolated HDL were loaded onto stain-free gel, which has a linear dynamic detection range and allows for protein detection on both gels and membranes along with the use of total protein measurement. Following gel electrophoresis with stain-free gels and in the presence of MW proteins (**A**), imaging using the Chemidoc MP System to check protein separation quality, the blot was incubated with primary antibodies against (**B**) SAA1, (**C**) fibrinogen, and (**D**) integrin β-3. Thereafter, the blot was incubated with the secondary antibody, followed by imaging and data analysis by Image Lab Software. Results are means ± SEM and originated from at least three subjects per group. Given the variability observed among individuals, the whole gels were shown according to the trend of the calculated average. **p < 0,01; ***p < 0,001.

**Figure 5:**
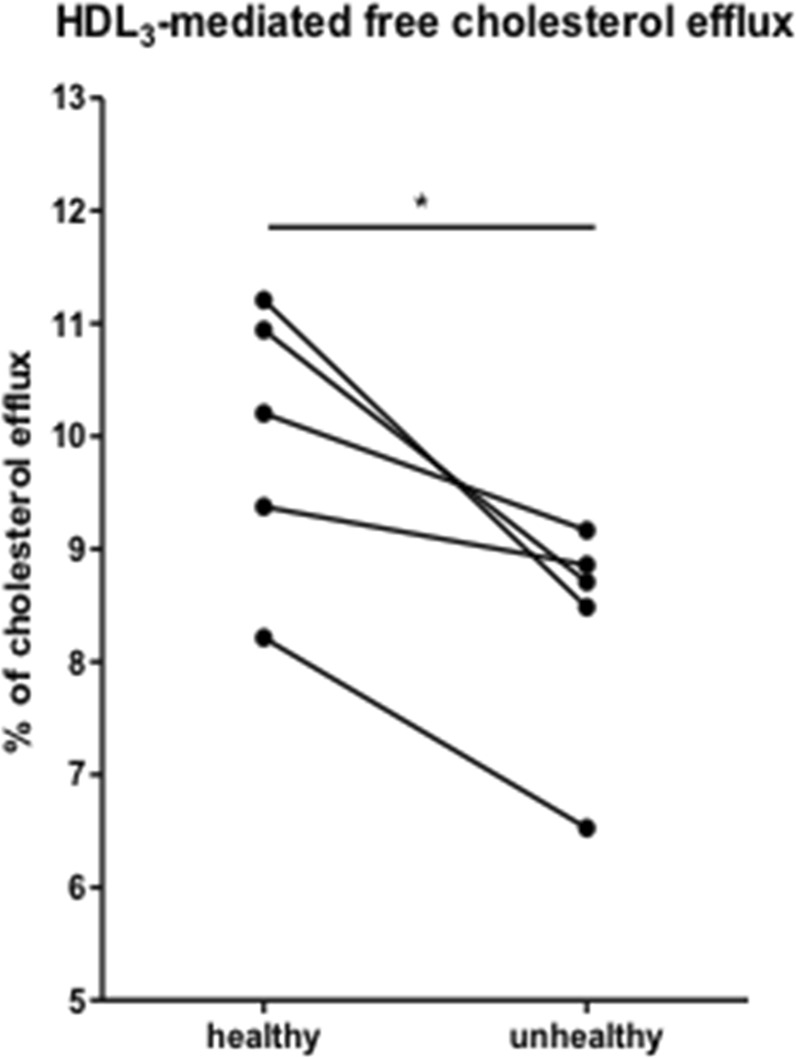
Effectiveness of HDL_3_ survivors in promoting cholesterol efflux. Differentiated Caco-2/15 cells were loaded with radiolabeled cholesterol by incubation for 24 h in 0.5 ml of supplemented RPMI with 5% v/v LPDS (lipoprotein-deficient serum) and 2.64 × 10^6^ dpm/ml [^14^C]-cholesteryl oleate oxLDL (50 μg Apo B/ml). After a 16-hour equilibration period of time without radioactivity, cells were washed with PBS and incubated with HDL_3_ (25 μg/ml) for 24 h. The media were centrifuged at 4000 g for 10 min to remove any suspended or dead cells and the cholesterol influx content was calculated by determining radioactivity in cell lysate. Values are means ± SEM for 5 separate survivors in each group (paired for age and sex). *p < 0,05

Biochemical validation of the MS was carried out data using the same HDL fractions employed for proteomics analysis. We especially selected 3 important proteins: (i) SAA that renders HDL dysfunctional, pro-inflammatory and inefficient to promote cholesterol efflux; (ii) fibrinogen that is prothrombotic; and (iii) integrin that contributes to the development of advanced fibrotic plaques and atherosclerosis. Figure 4 illustrates their protein expression provided by Western blot. Unhealthy survivors were characterized by a raised protein expression of SAA1, fibrinogen and integrin β-3, which confirms the results obtained by proteomics assessment. Healthy survivors and Controls displayed the same profile. Given the multiple changes in composition (Table 3) and alterations in proteomics (Fig. 3), we assumed that HDL from Unhealthy survivors are dysfunctional. To test this hypothesis, we examined the capacity of HDL_3_ to efflux cholesterol from cell line, HDL_3_ being mostly appropriate to elicit cellular cholesterol export (Fig. 5). Our findings showed the inefficiency of Unhealthy HDL to perform this biological function.

## Discussion

Our study on the HDLs of young ALL survivors indicates an altered metabolism and a shift in their proteome affecting specifically their anti-thrombotic, anti-apoptotic and anti-inflammatory capacities. These HDLs have an abnormal composition with a lower content of free and esterified cholesterol compared to controls. They also expressed some proteins not detectable in the control samples; most of them are either pro-thrombotic or pro-apoptotic. Finally, HDLs from unhealthy survivors expressed at least twice more of the pro-thrombotic and pro-inflammatory proteins SAA and fibrinogen compared to controls. The pro-atherogenic profile of the survivors was often worsened by dyslipidemia parameters such as high TG or high LDL-C. Although a few reports are available on HDLs in the context of inflammatory or auto-immune diseases, we are the first group to our knowledge to investigate HDL proteomics of cancer survivors, especially in the pediatric population.

HDLs are key players in the management of the body homeostasis and they help maintaining cardiometabolic health. Their anti-atherosclerosis action, through the promotion of cholesterol efflux and reverse transport to the liver leading to its final elimination (via bile acid conversion), is their most well-known and studied function^11,29^. However, HDLs have a pleiotropy of additional actions. Indeed, they display anti-inflammatory, anti-hypertensive, anti-apoptotic, antioxidative and anti-thrombotic actions, while also participating in the immune response^12,15,25,26,30–33^. The HDL multifunction is made possible by its high content of bioactive lipids and especially its numerous protein moieties. Undoubtedly, the appropriate composition of these components is primordial to the normal functioning of HDL since their imbalance is detrimental to cardiometabolic and cardiovascular health^34,35^.

Apparently, most subjects present low HDL-C levels when they receive a diagnosis of cancer^36–39^, which indicates a change in HDL metabolism due possibly to their proteomic content. Although the treatment and remission of cancer patients are usually associated with a rise in HDL-C levels^38^, a substantial proportion of survivors still exhibit low HDL-C levels even during the remission^37^. In addition to low HDL-C concentrations^36,40^, ALL survivors often present cardiometabolic derangements such as obesity, hypertension and dyslipidemia^40^. Previously, we demonstrated that altered lipoprotein composition characterized dyslipidemic ALL survivors^40^. Therefore, it is plausible that their HDLs may undergo inappropriate metabolism because of abnormal protein composition, thereby affecting their level of functionality. Surprisingly, this important aspect has never been studied. Since the functions of HDLs are strongly dependent on their protein content, we applied mass spectrometry techniques to investigate the proteome of HDLs isolated from childhood ALL survivors. Interest for proteomics is increasing and this straightforward approach is rapidly becoming a valuable tool for the confident identification of differentially expressed proteins. Applying the robust proteomics technology with high throughput capabilities would not only extend our knowledge and understanding of HDL metabolism in ALL, but it may also provide new potential diagnostic, prognostic or therapeutic biomarkers for the patients. Importantly, to control for the influence of biological, lifestyle and therapeutic factors on HDL composition, ALL survivors were paired for age, smoking and treatments.

### General composition of HDL

We report first that the HDLs from the unhealthy survivors of childhood ALL are depleted in free and esterified cholesterol, with no change in their other relative protein and lipid moieties. This depletion in cholesterol is not unexpected in the context of dyslipidemia characterizing ALL survivors as it has been reported in cardiometabolic disorders such as the metabolic syndrome^41^, obesity^42^ and diabetes^43^, or in complex pathologies including systemic lupus erythematosus^44^ and end-stage renal disease^34^. The type of cholesterol affected (free or esterified) differs between the studies. We are one of the few groups to report lowering of the two cholesterol forms^42,45^. A shift towards smaller HDLs is also observed by most of these groups^34,41,42,44,45^. However, they also report an enrichment of TG in the HDL, which we did not observe. Hence, cancer survivors seem to have a distinct pattern of HDL alterations, comprised of a diminution in the content of both forms of cholesterol (FC and EC), but no enrichment of TG. Our results are consistent with the shift towards smaller HDLs in pathological conditions, as demonstrated by a lower weight ratio in ALL Survivors^40^. The causes for low cholesterol in HDL of ALL survivors could be multiple. For example, limited LCAT activity^34,46^, poor cholesterol efflux from cells^42,46^ and abnormally high CETP activity^47^ have already been identified as causes of decreased HDL cholesterol content, which can render HDL inefficient in protecting against atherosclerotic plaque initiation and cardiovascular diseases development.

### Proteomic profiling by mass spectrometry

In a first step, we compared the high-resolution lipoproteomics of HDL from healthy controls to available findings in the literature in order to situate the robustness of our innovative approach. To this end, we selected 8 recent studies pertaining to HDL biology, which isolated HDLs by ultracentrifugation, adequately described their population, included a control arm, provided extensive data, separated the data of the controls and pathological subjects, and identified a significant amount of proteins^1,48,49^. The number of proteins identified in each study ranged from 49 to 190, while our own investigation revealed a total of 134 proteins in HDL. A total of 326 different proteins were reported in at least one of these studies or in our own, of which 171 (52,4%) were detected only once. We were able to highlight 7 new proteins in the HDL, namely actin alpha cardiac muscle 1, band 3 anion transport protein, homeobox protein Nkx-2.4, keratinocyte differentiation-associated protein, trypsin-2, trypsin-3 and UPF0669 protein C6orf120. Only 18 proteins were found in all the studies: 11 Apos (A-I, A-II, A-IV, C-I, C-II, C-III, D, E, J, L1, and M) along with complement C3, haptoglobin-related protein, phosphatidylcholine-sterol acyltransferase, serum paraoxonase/arylesterase 1, phospholipid transfer protein, serum amyloid A1/2, and vitronectin. Most of the HDL proteins (15/18) are related to lipid transport and metabolism. The only protein absent in our study but present in the other 8 investigations is alpha-1-acid glycoprotein 2. This protein is involved in the regulation of the immune system, the acute-phase response and the transport of diverse non-lipid molecules. Finally, of all the 115 proteins identified in at least 3 studies, 81 (70,4%) were detected in our work. The variations observed may be inherent to the different techniques and operators, the populations studied, and computational analyses.

### Imbalance of the anti-/pro-thrombotic equilibrium in HDL from survivors

In healthy individuals, HDLs are recognised to be anti-thrombotic, because they prevent platelet aggregation, regulate coagulation cascade, prolong the time of thrombus formation, and promote fibrin clot degradation. HDLs prevent platelet activation by inducing 2 of their potent inhibitors, e.g. prostacyclin PGI2 and NO (via upregulation of eNOS activity)^50^. HDLs also transport the protein platelet activating factor-acetylhydrolase (PAF-AH) which hydrolyses platelet activating factor. As to the fibrin clots, HDLs act both on their formation and degradation via reduction of thrombin formation and stimulation of plasmin generation^51–53^. Thrombin, the active form of prothrombin, is responsible for the cleavage of fibrinogen into fibrin, an insoluble fibre and the major clot component. Plasmin, the active form of plasminogen, is a key factor in fibrinolysis. In hypercoagulability states, anticoagulant functions are lowered, while the fibrinolysis is slowed down. In our study, of all the proteins that we identified, 46 (34%) were a part of the coagulation cascade, making it the third most frequent function, even surpassing lipid transport and metabolism. Thirteen HDL proteins expressed only by ALL Survivors were pro-thrombotic although half of the individuals exhibited the anti-thrombotic protein tissue factor pathway inhibitor. The wide variety of pro-thrombotic proteins found in these samples probably outweighs tissue factor pathway inhibitor’s effect. One of these pro-thrombotic proteins is thrombospondin-1, which is also pro-apoptotic and pro-inflammatory. It has been reported in HDLs from ACS patients, but not in stable CAD or healthy controls^48^. HDLs from patients with chronic heart failure were also not enriched in this protein^49^. It might be that the pro-thrombotic phenotype is associated with a more acute condition.

The analyses on the differential expression of the proteins can identify those that are reproducibly under- or overexpressed. In particular, the HDLs from the survivors displayed overexpression of many pro-thrombotic proteins, which could be consistent with the higher rate of thromboembolism in this population^4,54^. The HDLs from unhealthy survivors showed an overexpression of fibrinogen alpha chain (compared to healthy controls), and fibrinogen beta chain (compared to healthy survivors). Fibrinogen levels have been examined in cancer, but only in the serum, and usually at the diagnosis^55,56^. Even if a few groups investigated the effect of chemotherapy on its levels, no data was reported during treatment (>1 month). Normal plasma concentrations of fibrinogen have been found at diagnosis of childhood ALL^55–57^, but asparaginase administration made them drop by up to 75%^55,57^. Only one study reassessed the levels of serum fibrinogen and found that a 2-week asparaginase withdrawal was enough to normalize fibrinogen levels^57^. Another chemotherapy agent, methotrexate, did not impact fibrinogen levels in childhood ALL^58^. For now, the mechanisms for fibrinogen enrichment in HDLs from childhood ALL survivors are not clear. Since higher serum fibrinogen have been reported in populations of cancer survivors^37,59^, it is possible that this causes a significant binding to HDL. Since the HDL proteome has never been studied in cancer patients, we do not know whether this is exclusive to LLA survivors, only to pediatric cancer survivors, or common among all the cancer survivors.

### Imbalance of the anti-/pro-inflammatory equilibrium in HDL from survivors

In healthy individuals, HDLs are recognised to be anti-inflammatory, because they reduce the expression of several cell surface adhesion proteins, (intercellular adhesion molecule-1 (ICAM-1), vascular cell adhesion molecule-1 (VCAM-1), and E-selectin), and chemokines (monocyte chemotactic protein-1 (MCP-1)) by the endothelial cells^15,60^. These proteins are responsible for the recruitment and transmigration of macrophages in the intima, where they will absorb oxidized LDLs, transform into foam cells and begin the process of atherosclerosis. Apo A-I and the different phospholipids mediate most of this action^16,61^.

Systemic inflammation is seen in serious cardiovascular conditions, and associated with greater quantities of serum amyloid A (SAA), which can rise to a thousand times the normal levels^62^. SAA is a potent pro-inflammatory protein, able to stimulate the expression of ICAM/VCAM-1 and MCP-1^15,62,63^. HDLs in inflammation are often enriched in SAA, as seen in ACS^64^, STEMI^65^, CAD^64^, ESRD^15,66^ and psoriasis^35^ patients. SAA enrichment of HDL renders it dysfunctional, especially pro-inflammatory^15,60,64^, but also inefficient to promote cholesterol efflux^66^ and NO production^25^, in part because SAA is known to displace Apo A-I from the HDLs^35,62^. Although radiotherapy can cause a temporary rise of SAA and CRP in the blood circulation^67^, the effects of cancer and its treatments on SAA are unclear. It seems that SAA can rise in active untreated cancers and reaches the highest levels in metastatic or advanced malignancies^68^. However, it has not been studied whether chemotherapy or other treatments allow normalization of plasma SAA levels.

In our study, ALL survivors had a more inflammatory profile, as demonstrated by the higher serum levels of CRP and MDA compared to the controls. In line with published data, the HDLs from ALL survivors were also significantly enriched in SAA compared to the healthy controls. However, Apo A-I content was not altered in these samples. This combination of observations suggests that HDLs from survivors of pediatric cancers are less anti-inflammatory, if not downright pro-inflammatory. More studies are needed to investigate the functionality of SAA-enriched HDLs from cancer survivors.

We selected three proteins (SAA1, fibrinogen, integrin β-3) with different functions to further validate the proteomics data with classical immunoblotting. The data provide support for the increased expression recorded from proteomics analysis particularly in Unhealthy survivors. While the factors leading to the differences between the Healthy and Unhealthy survivors cannot be determined with our experimental design, it is possible that patient’s age at diagnosis and time of remission play a role, as they were the only different characteristics between groups. Given the numerous alterations characterizing their HDL, we hypothesize that this disturbed HDL is unable to fulfill their beneficial functions. Indeed, Unhealthy HDL_3_ were less effective in promoting cholesterol efflux, which is a key event in reverse cholesterol transport and protection against atherosclerosis.

It is important to note that our findings related to cardiometabolic disorders in cALL survivors are generalizable to European and non-European populations since our results are similar to those of the French L.E.A (Leucémie de l’Enfant et de l’Adolescent), the St. Jude Lifetime Cohort Study and different parts in the USA and the world^69–71^. Nevertheless, additional studies are required to determine whether HDL proteome are of general applicability.

### Conclusions and strengths/weaknesses of the study

This novel study describes for the first time the global HDL composition and reports the alterations in HDL proteome of childhood ALL survivors. Our findings not only emphasized a diminution in the content of both forms of cholesterol and the shift towards smaller HDLs, but they also underlined an imbalance of anti-/pro-inflammatory and an anti-/pro-thrombotic equilibrium in HDL. In these conditions, pro-inflammatory and pro-thrombotic protein content in HDL, will amplify MetS disorders, creating vicious cycle and magnifying the risks for cardiovascular diseases. The limitations of this exploratory work are its limited sample size and descriptive nature. Furthermore, we do not have any information on the post-translational modifications on the proteins, which could strongly affect the functionality of the lipoproteins. Although more work is needed to validate the functionality of HDLs, the present findings may be relevant to the field of cancer survivorship in providing potential diagnostic, prognostic or therapeutic biomarkers of HDL functionality and metabolism in ALL.

## Methods

### Study participants

Participants for this study were recruited as part of the PETALE initiative at the Sainte-Justine University Hospital Center (UHC) in Montreal. The PETALE program aims to identify biomarkers associated to the development of late effects in survivors of pediatric ALL. The PETALE cohort is comprised exclusively of childhood ALL survivors of European descent from the province of Québec. PETALE participants were diagnosed between 1987 and 2010, under 19 at diagnosis, and treated at Sainte-Justine University Hospital Center (UHC) with the Dana-Farber Cancer Institute protocols^72^. Exclusion criteria included patients treated with hematopoietic stem cell transplantations, or those with leukemia relapses or secondary cancers^73^. Recruitment was held between January 2012 and September 2015. The study was approved by the Sainte-Justine UHC Institutional Review Board, and all interventions were carried out in respect of the Declaration of Helsinki. Written informed consent from the participant and/or the legal guardian was obtained prior to participation.

### Clinical, biochemical and anthropometric data

Height, weight and waist circumference were documented for every participant. BMI was calculated as weight divided by height squared (Kg/m^2^). Blood pressure was measured as per Hypertension Canada’s guidelines^74^. Information on smoking habits was obtained from a self-reported questionnaire. The date of diagnostic, total radiotherapy dose and end-of-treatment date were collected directly from patients’ charts. Age at diagnosis and event-free remission were then computed. Total cholesterol (TC), HDL-C and triglycerides (TG) were enzymatically determined with a Synchron LX®20 (Beckman Coulter, USA) using Beckman Instrument reagents. LDL-cholesterol (LDL-C) was calculated using the Friedewald equation^75^, while non-HDL-cholesterol (non-HDL-C) was defined as: CT - (HDL-C). Blood glucose was assessed with the glucose oxidase technique and insulin with an Access Immunoassay System (Beckman Coulter, USA). Finally, participants were submitted to an oral glucose tolerance test (OGTT), which evaluated their glycemic response to 75 g glucose at 30, 60 and 120 minutes^73^. For this study, participants were classified as «metabolically healthy» (Healthy) or «metabolically unhealthy» (Unhealthy). The Healthy subjects did not present any symptom of the metabolic syndrome, whereas the Unhealthy had at least a low HDL-C (<1.03 mmol/L for men, or <1.3 mmol/L for women)^76^ with or without another metabolic disorder such as hypertension, obesity, dyslipidemia and glucose intolerance. Cumulative doses of corticosteroids were calculated as the sum of prednisone-equivalent, body-surface area-normalized (mg/m^2^) dexamethasone, methylprednisolone, prednisolone, and prednisone doses received during the three phases of the chemotherapy treatment (induction, intensification, and consolidation). Participants were individually selected in order to match them for age and gender with survivors displaying the opposite phenotype, which resulted in the identification of 8 pairs of healthy-unhealthy survivors, in addition to 8 control subjects for normal references.

### HDL isolation

Plasma HDLs were isolated as described in previous work^40^. Briefly, antecubital venous blood was obtained after an overnight fast in EDTA-coated tubes (1 g EDTA/L). Plasma was separated from blood by a 20-min low-speed centrifugation at 4 °C. BHT (0.01%) and sodium azide (NaN3, 0.01%) were added to preserve the samples. Total HDLs (δ = 1.09–1.21 g/ml) were isolated by density gradient ultracentrifugation using KBr and an Optima LE-80 ultracentrifuge (Beckman Coulter, USA) with a 70.1ti rotor (110 000 g, 4 °C, 48 h). Samples were desalted by a 24-h dialysis against a 0.15 M NaCl- 0.3 mM EDTA buffer (pH 7,4) and kept at −80 °C until assayed.

### HDL composition

HDL composition is expressed as percent of esterified cholesterol (EC), free cholesterol (FC), phospholipids (PL), TG and total proteins as described in^40^. CT, CL and TG were quantified using enzymatic colorimetric commercial kits (Wako Pure Chemical Industries, Japan, and Randox TRIGS, United Kingdom). CE was estimated as (CT – CL) X 1.67. The Bradford technique (Bio-Rad reagent) was applied to determine the protein concentration^77^, whereas phospholipids were quantified with the Bartlett method^78^. The lipoprotein weight ratio, which gives an estimation of its size, was computed as the ratio of the components in the core of the particle to the components on its surface: (TG + EC)/(PL + FC + proteins)^79^.

### HDL delipidation

For the mass spectrometry analysis, lipid-extracted HDL fractions were employed since phospholipids are known to interfere with the ionisation process, reduce the signal and produce low-quality results^80^. To one volume of HDL, 5 volumes of a mix of ethanol: ethyl ether (3:1) were added, thoroughly vortexed and left at −20 °C overnight. The supernatant was separated in a 25-min low-speed centrifugation at −20 °C, then removed and replaced with 2 volumes of the initial mix. Following a second 4 hour-incubation at −20 °C, the supernatant was isolated in the same conditions and removed completely. Protein pellets were dried under nitrogen, dissolved in saline of physiological density (1.006 g/ml) and homogenised.

### Mass spectrometry analyses and protein quantification

The mass spectrometry analyses were carried out at the Institute for Research in Immunology and Cancer (Université de Montréal, Canada) on 25 µg of proteins from delipidated HDL. Sample reduction was performed by adding 50 µl of 5 mM TCEP in 100 mM ammonium bicarbonate to the dry sample. Alkylation was performed by adding 50 µl of chloroacetamide 20 mM with ammonium bicarbonate 100 mM. Trypsin (1 µg) was added and the digestion was performed for 8 h at 37 °C. Samples were loaded and separated on a home-made C_18_ analytical column (15 cm * 150 µm i.d.) with a 116-min gradient from 0–30% acetonitrile (0.2% FA) and a 600 nl/min flow rate on a Easy nLC (Dionex) connected to a Q-Exactive HF (Thermo Fisher Scientific). Each full MS spectrum acquired with a 60,000 resolution was followed by up to 20 MS/MS spectra, where the 20 most abundant multiply charged ions were selected for MS/MS sequencing. Peptides were identified using Peaks 8.0 (Bioinformatics Solution Inc.) and peptide sequences were blasted against the Human Uniprot database. Tolerance was set at 10 ppm for precursor and 0.01 Da for-fragment ions for protein identification and 7ppm and 2 min for peptide alignment and profiling. FDR threshold was set to 0.01. For the post-translational modification of proteins, occurrence of carbamidomethylation (C), oxidation (M), deamidation (NQ) was considered. Volcano plots were made with Perseus 1.5.0.30.

### Statistical analyses

Results are presented as means ± SEM. The distributions were systematically tested for normality before the appropriate tests were applied. The parametric (Student t-test, Student t-test for paired data, repeated one-way ANOVA with Newman-Keuls post-hoc analysis) and non-parametric tests (Mann Whitney’s U test, Wilcoxon’s, or Friedman’s with Dunn’s post-hoc analysis) were performed using Prisme 5.01 (GraphPad Software, USA). A p-value of 0.05 was considered significant.

## Supplementary information

Supplementary data
