# Supplementary material for: Altered proteome of high-density lipoproteins from paediatric acute lymphoblastic leukemia survivors
Source: Sci Rep. 2019 Mar 12;9:4268. doi: 10.1038/s41598-019-40906-x (PMC6414624; doi:10.1038/s41598-019-40906-x)
Supplement: Supplementary file 1 — Supplementary data [file 41598_2019_40906_MOESM1_ESM.pdf]

Altered proteome of high-density lipoproteins from paediatric acute lymphoblastic leukemia survivors

Maryse Fournier[1,2], Eric Bonneil[3], Carole Garofalo[1], Guy Grimard[4], Caroline Laverdière[1,4], Maja Krajinovic[1,4], Simon Drouin[1], Daniel Sinnett[1,4], Valérie Marcil[1,2] and Emile Levy[1,2*]

[1]Research Centre, Sainte-Justine University Hospital Health Center, Departments of [2]Nutrition and [4]Pediatrics, Université de Montréal, Montreal, Quebec, Canada, H3T 1C5.

[3]Institute for Research in Immunology and Cancer, Université de Montréal, Montréal, QC, Canada, H3C 3J7

Address for correspondence: *Dr. Emile Levy
GI-Nutrition Unit
CHU Sainte-Justine
3175 Sainte-Catherine Road
Montreal, Quebec, Canada, H3T 1C5
Tel.: (514) 345-7783
Fax: (514) 345-4999
E-mail: emile.levy@recherche-ste-justine.qc.ca

Table S1: Protein identification and distribution among the ALL and Control groups

	Protein	Accession number	Number of exclusive unique peptides	% of sequence coverage	Group distribution				
					C	ALL survivors			Total
						All	H	U	
1	Alpha-1-antitrypsin	P01009	448	48.2 ± 6.7	8	16	8	8	24
2	Alpha-2-HS-glycoprotein	P02765	192	26.0 ± 4.9	8	16	8	8	24
3	Apolipoprotein A-I	P02647	3633	91.7 ± 1.2	8	16	8	8	24
4	Apolipoprotein A-II	P02652	1074	77.2 ± 3.9	8	16	8	8	24
5	Apolipoprotein A-IV	P06727	1030	75.8 ± 8.1	8	16	8	8	24
6	Apolipoprotein B	P04114	2635	27.8 ± 11.8	8	16	8	8	24
7	Apolipoprotein C-I	P02654	540	61.6 ± 7.1	8	16	8	8	24
8	Apolipoprotein C-II	P02655	257	76.2 ± 8.9	8	16	8	8	24
9	Apolipoprotein C-III	P02656	381	76.6 ± 2.5	8	16	8	8	24
10	Apolipoprotein C-IV	P55056	87	24.5 ± 7.5	8	16	8	8	24
11	Apolipoprotein D	P05090	337	43.6 ± 5.6	8	16	8	8	24
12	Apolipoprotein E	P02649	895	81.9 ± 5.8	8	16	8	8	24
13	Apolipoprotein F	Q13790	130	18.7 ± 3.8	8	16	8	8	24
14	Apolipoprotein J	P10909	409	36.1 ± 3.8	8	16	8	8	24
15	Apolipoprotein L1	O14791	446	39.0 ± 6.1	8	16	8	8	24
16	Apolipoprotein M	O95445	428	62.7 ± 10.4	8	16	8	8	24
17	Complement C3	P01024	284	9.7 ± 3.3	8	16	8	8	24
18	Fibrinogen alpha chain	P02671	601	38.7 ± 8.8	8	16	8	8	24
19	Fibrinogen beta chain	P02675	232	17.0 ± 5.6	8	16	8	8	24
20	Haptoglobin-related protein	P00739	281	36.0 ± 13.1	8	16	8	8	24
21	Hemoglobin subunit beta	P68871	225	67.7 ± 11.7	8	16	8	8	24
22	Phosphatidylcholine-sterol acyltransferase (LCAT)	P04180	188	24.1 ± 4.0	8	16	8	8	24
23	Serum paraoxonase/ arylesterase 1 (PON1)	P27169	470	65.2 ± 9.6	8	16	8	8	24
24	Serum paraoxonase/ lactonase 3 (PON3)	Q15166	165	33.6 ± 8.7	8	16	8	8	24
25	Retinol-binding protein 4	P02753	131	33.2 ± 17.4	8	16	8	8	24

26	Serum amyloid A1	P0DJI8	202	66.3 ± 13.0	8	16	8	8	24
27	Serum amyloid A2	PODJI9	59	47.1 ± 15.8	8	16	8	8	24
28	Serum amyloid A4	P35542	334	62.3 ± 6.7	8	16	8	8	24
29	Serum albumin	P02768	1623	82.8 ± 5.0	8	16	8	8	24
30	Vitamin D-binding protein	P02774	205	24.0 ± 9.1	8	16	8	8	24
31	Vitronectin	P04004	138	15.2 ± 4.0	8	16	8	8	24
32	Cholesteryl ester transfer protein (CETP)	P11597	78	9.5 ± 2.8	8	15	8	7	23
33	Complement C4-B	P0C0L5	200	7.5 ± 3.7	7	16	8	8	23
34	Platelet basic protein	P02775	129	41.9 ± 7.9	7	16	8	8	23
35	Phospholipid transfer protein (PLTP)	P55058	167	18.0 ± 7.3	8	15	8	7	23
36	Actin, cytoplasmic 1	P60709	217	39.5 ± 11.2	8	14	7	7	22
37	Anthrax toxin receptor 2	P58335	59	7.4 ± 3.6	8	14	7	7	22
38	Prenylcystein oxidase 1	Q9UHG3	185	22.1 ± 6.7	7	15	7	8	22
39	Transthyretin	P02766	146	62.0 ± 9.4	7	15	7	8	22
40	Hemoglobin subunit alpha	P69905	109	44.1 ± 14.9	5	15	8	7	20
41	Platelet factor 4	P02776	78	39.2 ± 12.5	6	14	7	7	20
42	Protein MENT	Q9BUN1	49	9.1 ± 1.5	6	14	8	6	20
43	Apolipoprotein H	P02749	80	15.9 ± 7.4	6	13	6	7	19
44	Apolipoprotein (a)	P08519	431	6.7 ± 3.8	6	12	6	6	18
45	Beta-Ala-His dipeptidase	Q96KN2	70	12.9 ± 5.2	6	12	6	6	18
46	Keratinocyte differentiation-associated protein	P60985	31	27.4 ± 17.1	7	11	5	6	18
47	Proactivator polypeptide	P07602	28	4.5 ± 1.0	5	13	7	6	18
48	Haptoglobin	P00738	26	19.3 ± 7.7	4	13	7	6	17
49	Ig alpha-1 chain C region	P01876	58	15.3 ± 4.7	6	11	4	7	17
50	Keratin, type II cytoskeletal 1	P04264	183	18.5 ± 11.3	7	10	4	6	17
51	Keratin, type I cytoskeletal 9	P35527	162	21.7 ± 9.6	5	11	4	7	16
52	Apolipoprotein A-V	Q6Q788	51	17.6 ± 7.2	4	11	7	4	15
53	Fibrinogen gamma chain	P02679	44	11.3 ± 3.5	3	12	6	6	15

54	Protein AMBP	P02760	33	8.8 ± 2.6	4	11	6	5	15
55	Actin, alpha cardiac muscle 1	P68032	18	23.1 ± 4.8	3	11	5	6	14
56	Band 3 anion transport protein	P02730	41	7.1 ± 3.2	2	10	4	6	12
57	Filamin-A	P21333	58	3.7 ± 2.0	2	10	5	5	12
58	Gelsolin	P06396	27	6.1 ± 2.1	3	8	2	6	11
59	Proprotein convertase subtilisin/kexin type 9	Q8NBP7	17	3.2 ± 0.5	5	6	4	2	11
60	Carbonic anhydrase 6	P23280	25	11.9 ± 2.8	4	5	2	3	9
61	Inter-alpha-trypsin inhibitor heavy chain H4	Q14624	56	8.8 ± 2.3	4	5	3	2	9
62	Talin-1	Q9Y490	44	3.3 ± 2.1	3	6	3	3	9
63	UPF0669 protein C6orf120	Q7Z4R8	13	18.0 ± 7.8	4	5	2	3	9
64	Complement component C9	P02748	20	4.8 ± 1.2	2	6	4	2	8
65	Serotransferrin	P02787	66	13.8 ± 10.6	1	7	4	3	8
66	Tissue factor pathway inhibitor	P10646	14	13.4 ± 4.5	0	8	2	6	8
67	Angiotensinogen	P01019	17	6.7 ± 1.2	1	6	3	3	7
68	Cathelicidin antimicrobial peptide	P49913	24	20.0 ± 3.1	3	4	2	2	7
69	Homeobox protein Nkx-2.4	Q9H2Z4	18	2.8 ± 0.0	3	4	1	3	7
70	Multimerin-2	Q9H8L6	13	3.8 ± 0.4	0	7	4	3	7
71	Plasma protease C1 inhibitor	P05155	10	4.1 ± 1.0	2	5	3	2	7
72	Fibronectin	P02751	9	1.6 ± 0.7	0	6	3	3	6
73	Keratin type 1, cytoskeletal 10	P13645	16	14.7 ± 9.9	3	3	1	2	6
74	Alpha-2-antiplasmin	P08697	13	7.4 ± 1.8	3	2	1	1	5
75	Beta-2-microglobulin	P61769	13	23.3 ± 12.6	1	4	1	3	5
76	Complement factor D	P00746	10	16.0 ± 1.6	1	4	2	2	5
77	Fibroblast growth factor-binding protein 2	Q9BYJ0	10	14.6 ± 5.4	1	4	2	2	5
78	Integrin alpha-IIb	P08514	21	7.2 ± 3.1	1	4	2	2	5

79	14-3-3 protein zeta/delta	P63104	14	19.5 ± 5.0	0	4	2	2	4
80	HLA class I histocompatibility antigen, A-24 alpha chain	P05534	33	33.5 ± 8.3	1	3	1	2	4
81	Ig kappa chain C region	P01834	7	26.3 ± 5.8	1	3	2	1	4
82	Integrin beta-1	P05556	7	3.8 ± 0.8	0	4	4	0	4
83	Integrin beta-3	P05106	15	6.7 ± 2.0	0	4	2	2	4
84	Keratin, type II cytoskeletal 2 epidermal	P35908	18	14.1 ± 8.5	2	2	1	1	4
85	Kininogen-1	P01042	11	4.5 ± 1.0	3	1	0	1	4
86	Profilin-1	P07737	10	21.8 ± 8.8	2	2	2	0	4
87	Pulmonary surfactant-associated protein B	P07988	15	22.5 ± 10.5	1	3	2	1	4
88	SPARC-like protein 1	Q14515	8	5.9 ± 1.3	0	4	3	1	4
89	Hemoglobin subunit delta	P02042	6	57.0 ± 10.7	0	3	1	2	3
90	Hemopexin	P02790	6	10.2 ± 0.5	1	2	1	1	3
91	Indian hedgehog protein	Q14623	8	14.3 ± 3.6	0	3	1	2	3
92	Myosin-9	P35579	22	5.8 ± 5.5	0	3	1	2	3
93	Platelet-activating factor acetylhydrolase (PAF-AH)	Q13093	7	6.8 ± 1.8	2	1	1	0	3
94	Protein Z-dependent protease inhibitor	Q9UK55	5	8.5 ± 3.3	1	2	2	0	3
95	Ras-related protein Rap-1b	P61224	6	12.0 ± 1.3	0	3	3	0	3
96	Thrombospondin-1	P07996	12	4.9 ± 2.1	0	3	2	1	3
97	CD99 antigen	P14209	4	23.0 ± 17.1	0	2	0	2	2
98	Cystatin-C	P01034	6	29.0 ± 4.0	1	1	0	1	2
99	Dermcidin	P81605	3	25.0 ± 6.0	0	2	0	2	2
100	Glyceraldehyde-3-phosphate dehydrogenase	P04406	7	18.0 ± 6.0	0	2	2	0	2
101	Ig gamma-1 chain C region	P01857	3	7.4 ± 2.6	0	2	2	0	2
102	Keratin, type 1 cytoskeletal 14	P02533	3	7.7 ± 1.6	2	0	0	0	2

103	L-selectin	P14151	7	8.8 ± 0.0	1	1	1	0	2
104	Pigment epithelium-derived factor	P36955	7	8.6 ± 5.5	0	2	1	1	2
105	Plasminogen	P00747	5	5.9 ± 0.6	1	1	0	1	2
106	Platelet glycoprotein Ib beta chain	P13224	4	5.4 ± 0.0	1	1	1	0	2
107	Trypsin-3	P35030	4	11.5 ± 1.5	1	1	1	0	2
108	Tubulin alpha-4A chain	P68366	7	12.0 ± 6.0	0	2	2	0	2
109	Alpha-2-macroglobulin	P01023	5	6.5	1	0	0	0	1
110	Alpha-actinin-1	P12814	6	9.2	0	1	1	0	1
111	Angiopoietin-related protein 3	Q9Y5C1	2	8.0	1	0	0	0	1
112	Ankyrin-1	P16157	2	2.7	0	1	0	1	1
113	Anthrax toxin receptor 1	Q9H6X2	2	5.7	1	0	0	0	1
114	C4b-binding protein alpha chain	P04003	5	14.0	0	1	0	1	1
115	Coagulation factor XIII A chain	P00488	3	5.6	0	1	1	0	1
116	Cofilin-1	P23528	2	27.0	0	1	1	0	1
117	Fructose-bisphosphate aldolase A	P04075	2	13.0	0	1	1	0	1
118	Glycogen phosphorylase, muscle form	P11217	5	11.0	0	1	0	1	1
119	GTP-binding protein SAR1a	Q9NR31	2	27.0	0	1	1	0	1
120	Integrin-linked protein kinase	Q13418	3	11.0	0	1	1	0	1
121	Leucine-rich alpha-2-glycoprotein	P02750	3	17.0	0	1	1	0	1
122	Multimerin-1	Q13201	2	2.5	0	1	1	0	1
123	Myosin light polypeptide 6	P60660	2	15.0	0	1	1	0	1
124	Peroxyredoxin-6	P30041	2	15.0	0	1	1	0	1
125	Phosphatidylinositol-glycan-specific phospholipase D	P80108	2	3.8	0	1	1	0	1
126	Pleckstrin	P08567	3	19.0	0	1	1	0	1
127	Pyruvate kinase isozymes M1/M2	P14618	2	6.2	0	1	1	0	1

128	RUN domain-containing protein 3A	Q59EK9	2	1.7	0	1	0	1	1
129	Sonic hedgehog protein	Q15465	2	6.7	1	0	0	0	1
130	Transitional endoplasmic reticulum ATPase	P55072	2	4.3	0	1	1	0	1
131	Tropomyosin alpha-3 chain	P06753	2	14.0	0	1	1	0	1
132	Trypsin-2	P07478	2	17.0	1	0	0	0	1
133	Tubulin beta-1 chain	Q9H4B7	3	12.0	0	1	1	0	1
134	Tubulin beta-2A chain	Q13885	2	11.0	0	1	1	0	1

Proteins from delipidated HDLs were identified using liquid chromatography-tandem mass spectrometry. Identification thresholds were: FDR <1%, minimum 2 peptides in a single sample. Results are presented as Mean ± SEM. Controls (C, n=8), Healthy (H, n=8), Unhealthy (U, n=8)

Supplementary Figure S3: Volcano plot representations of the proteins statistically differently expressed between Women groups. **(A)** Controls vs Unhealthy (women only), **(B)** Controls vs Healthy (women only). X-axis represents the variation in expression [$\log_2$(fold change)], y-axis represents the statistical significance [-log(p-value)]. Proteins with coordinates (x,y) where x is <-1 or >1 and y is > 1,3 are statistically reproducibly halved or doubled in a group of samples.

Supplementary Figure S4: Volcano plot representations of the proteins statistically differently expressed between Men groups. (A) Controls vs Unhealthy, **(B)** Controls vs Healthy. X-axis represents the variation in expression [$\log_2$(fold change)], y-axis represents the statistical significance [-log(p-value)]. Proteins with coordinates (x,y) where x is <-1 or >1 and y is > 1,3 are statistically reproducibly halved or doubled in a group of samples.

Figure S3

A

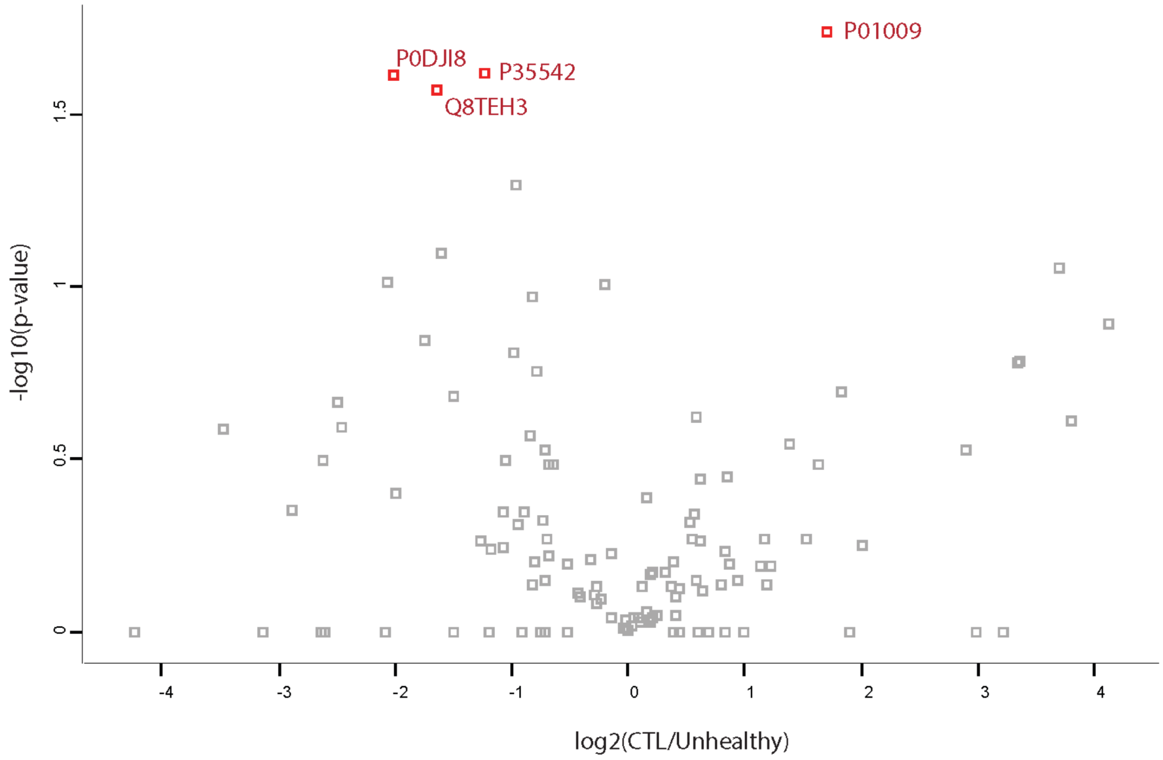
P01009
P0DJI8
P35542
Q8TEH3
-log10(p-value)
log2(CTL/Unhealthy)
0
0.5
1
1.5
-4
-3
-2
-1
0
1
2
3
4

B

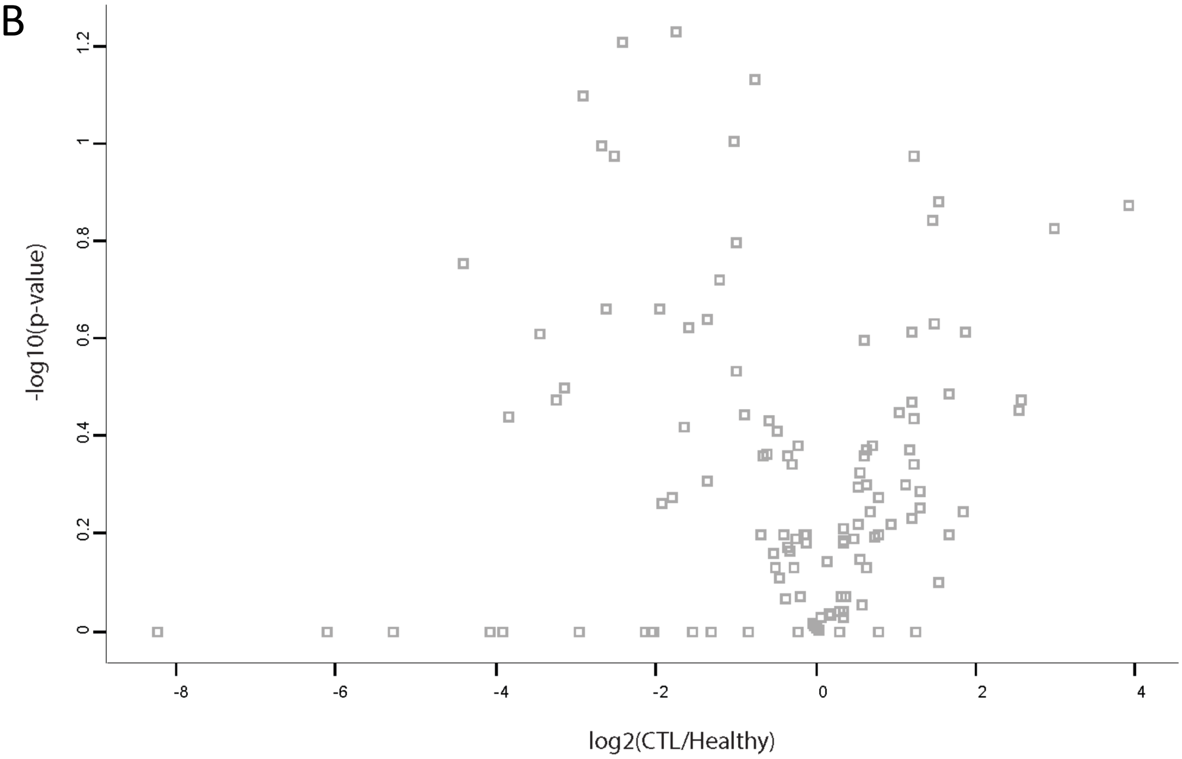
-log10(p-value)
log2(CTL/Healthy)
0
0.2
0.4
0.6
0.8
1
1.2
-8
-6
-4
-2
0
2
4

Figure S4

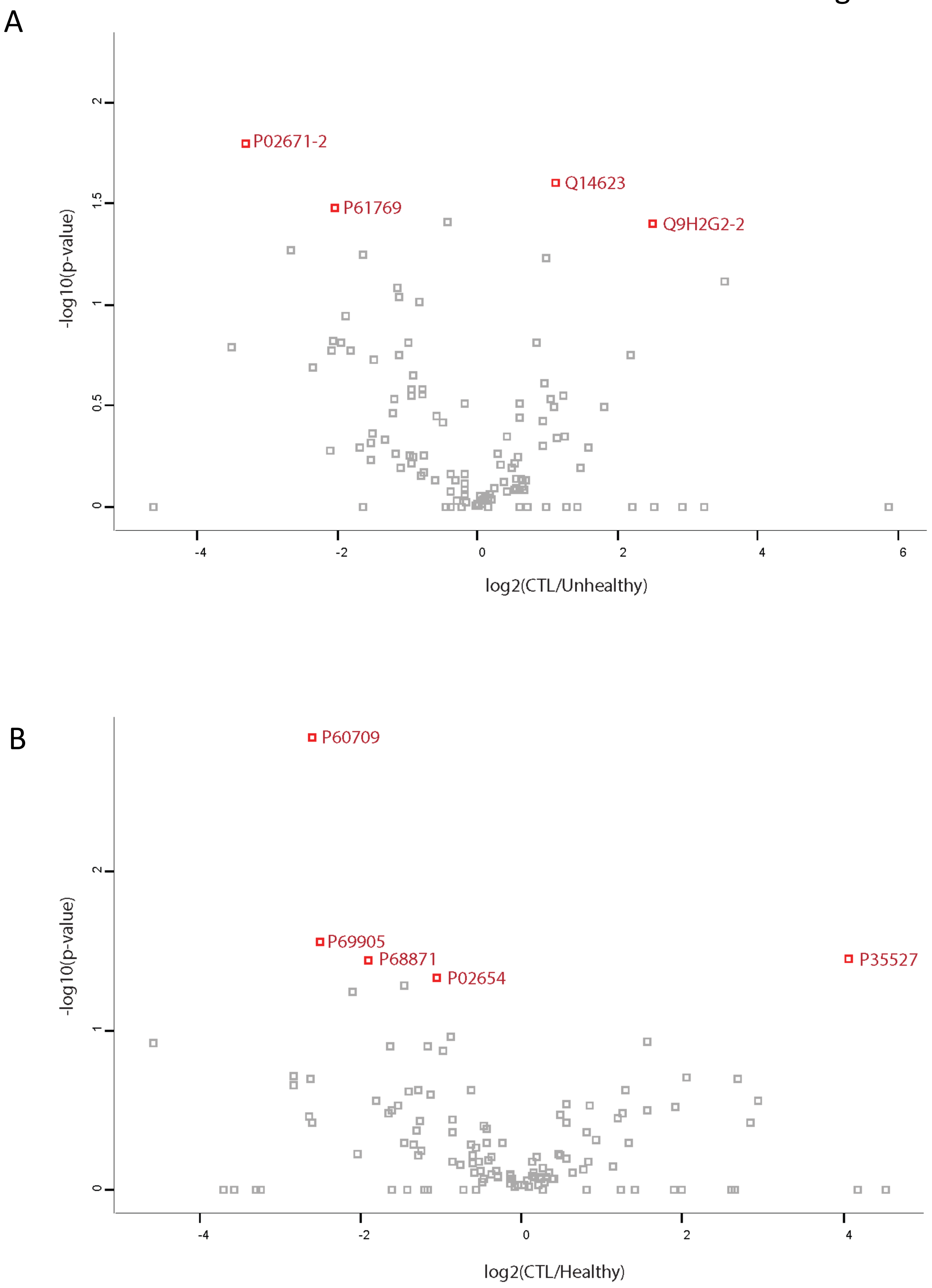
A
P02671-2
Q14623
P61769
Q9H2G2-2
-log10(p-value)
log2(CTL/Unhealthy)
B
P60709
P69905
P68871
P02654
P35527
-log10(p-value)
log2(CTL/Healthy)
